# Development of Alkylthiazole-Based Novel Thermoelectric Conjugated Polymers for Facile Organic Doping

**DOI:** 10.3390/nano13071286

**Published:** 2023-04-06

**Authors:** Junho Kim, Eui Hyun Suh, Kyumin Lee, Gyuri Kim, Hansu Kim, Jaeyoung Jang, In Hwan Jung

**Affiliations:** 1 Department of Energy Engineering, Hanyang University, Seoul 04763, Republic of Korea; 2 Department of Organic and Nano Engineering, and Human-Tech Convergence Program, Hanyang University, 222 Wangsimni-ro, Seongdong-gu, Seoul 04763, Republic of Korea

**Keywords:** organic thermoelectric devices, conjugated polymers, organic dopant, sequential doping, planar alkylthiazole building blocks

## Abstract

In this study, we developed two novel conjugated polymers that can easily be doped with F4TCNQ organic dopants using a sequential doping method and then studied their organic thermoelectric (OTE) properties. In particular, to promote the intermolecular ordering of OTE polymers in the presence of the F4TCNQ dopant, alkylthiazole-based conjugated building blocks with highly planar backbone structures were synthesized and copolymerized. All polymers showed strong molecular ordering and edge-on orientation in the film state, even in the presence of the F4TCNQ organic dopant. Thus, the sequential doping process barely changed the molecular ordering of the polymer films while making efficient molecular doping. In addition, the doping efficiency was improved in the more π-extended polymer backbones with thienothiophene units due to the emptier space in the polymer lamellar structure to locate ionized F4TCNQ. Moreover, the study of organic thin-film transistors (OTFTs) revealed that higher hole mobility in OTFTs was the key to increasing the electrical conductivity of OTE devices fabricated using the sequential doping method.

## 1. Introduction

Thermoelectric (TE) generators are renewable energy devices that convert wasted thermal energy into electrical energy [1,2,3,4,5,6]. The energy conversion efficiency of TE devices is determined by the dimensionless figure of merit, ZT = S^2^σ/κ, where S denotes the Seebeck coefficient, σ denotes the electrical conductivity, and κ denotes the thermal conductivity; thus, high S and σ values with a low κ value are required to improve TE performance. However, because there is a substantial trade-off relationship between S and σ values, optimization of S^2^σ, known as the power factor (PF), is the key to maximizing energy conversion efficiency [7,8,9].

Among the various types of TE materials [10,11,12,13,14], such as Bi_2_Te_3_, SnSe, single-wall carbon nanotubes (SWCNT), and graphene, organic materials based on conjugated polymers have unique advantages due to their low κ values, semi-transparency, low weight, solution-processibility, high energy conversion efficiency over a temperature range near room temperature (RT), etc. [15,16,17,18,19]. However, because hopping-type charge carrier transport is dominant in conjugated polymers, they have low charge carrier mobilities and a small mean free path in electronic devices, resulting in low electrical conductivity [20,21,22,23,24,25]. Various molecular doping methods that increase the charge carrier density have been developed to overcome this critical problem, and they have become essential processes for organic TE (OTE) devices [26,27,28]. FeCl_3_ is widely used for p-doping of conjugated polymers, owing to its strong oxidizing properties, and it has successfully increased the electrical conductivity of polymers in OTE devices [29,30,31]. However, their high reactivity under ambient conditions reduces the doping stability of OTE devices [32,33,34]. As an alternative method, organic dopants, such as 2,3,5,6-tetrafluoro-7,7,8,8-tetracyanoquinodimethane (F4TCNQ) and 2,3-dichloro-5,6-dicyano-1,4-benzoquinone (DDQ), which are stable in ambient environments, have received significant attention for use in OTE devices [35,36,37,38,39,40]. Chabinyc et al. reported several doping methods for OTE devices using F4TCNQ. Typical solution-doping showed a PF of 0.42 μW m^−1^ K^−2^ due to a smaller orientational correlation length, but vapor-annealing doping significantly increased the PF up to 120 μW m^−1^ K^−2^ [41]. Wang et al. developed benzo [1,2-b:4,5-b′]dithiophene-based conjugated polymers and studied their TE properties using DDQ and F4TCNQ organic dopants. A maximum PF of 0.21 μW m^−1^ K^−2^ was achieved by solution-doping with F4TCNQ [42]. Our research group developed 4H-cyclopenta[2,1-b:3,4-b’]dithiophene (CPDT)-based polymers and studied their thermoelectric properties by solution doping with F4TCNQ. The highest PF of 23.7 μW m^−1^ K^−2^ was achieved by controlling the alkyl side chains of the polymers [43,44]. However, owing to the relatively low doping efficiency of F4TCNQ and DDQ, these polymers usually do not reach their optimum doping levels to maximize PFs.

**Scheme 1:**
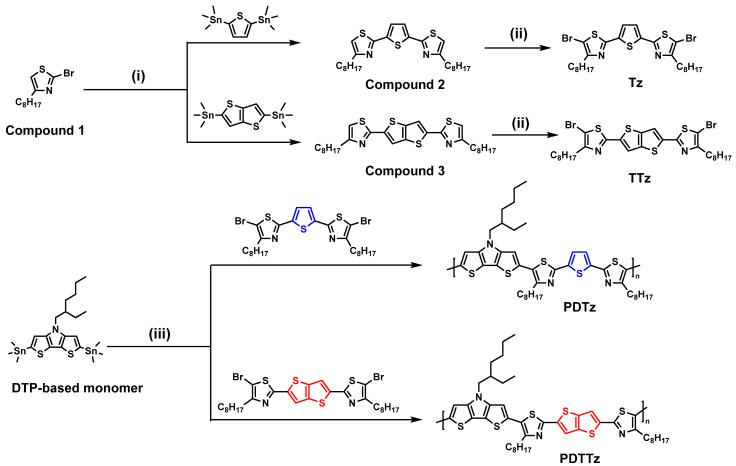
Synthesis of monomers and polymers. Reaction conditions: (**i**) Pd_2_(dba)_3,_ P(o-tolyl)_3_, toluene, 110 °C, procedure 24 h (**ii**) DMF, NBS, RT, overnight (**iii**) Pd(PPh_3_)_4_, toluene, 110 °C.

In this study, we synthesized 4H-dithieno[3,2-b:2′,3′-d]pyrrole (DTP)-based conjugated polymers that can be easily doped with F4TCNQ via a sequential doping method in which the F4TCNQ solution is spin-coated on pristine polymer films. DTP is a fused aromatic ring composed of two strong electron-donating thiophene rings and one pyrrole ring, imparting one of the strongest reported electron-donating properties to conjugated polymers [45,46,47]. Thus, we expected that the DTP-based polymers could be easily doped with an electron-withdrawing F4TCNQ dopant via a sequential doping method. More importantly, to promote the intermolecular ordering of DTP-based polymers in the presence of the F4TCNQ dopant, novel alkylthiazole-based conjugated building blocks with highly planar backbone structures (Tz and TTz) were also synthesized, as shown in Scheme 1. The lone pairs of nitrogen atoms on 4-octylthiazole interact with adjacent hydrogen atoms of thiophene and thienothiophene, which reduces the steric repulsion between aromatic rings and makes good π-π stacking of the conjugated polymer backbones [48]. Finally, two TE polymers were developed by Stille copolymerization of DTP-based monomers with Tz and TTz comonomers, and the resulting polymers were named PDTz and PDTTz, respectively.

The hole-transporting characteristics of the PDTz and PDTTz polymers were evaluated by fabricating organic field-effect transistor (OFET) devices; PDTTz showed twice as high hole mobility as PDTz. The greater number of π-extended polymeric backbones and aligned alkyl side chains in PDTTz resulted in better molecular ordering and higher hole mobility in the film states. The TE properties of the polymer films were investigated using sequential doping with an F4TCNQ solution. The PDTz and PDTTz devices showed the highest PFs of 0.18 ± 0.01 at a dopant concentration of 0.5 mg mL^−1^ and 0.48 ± 0.02 μW m^−1^ K^−2^ at a dopant concentration of 1.5 mg mL^−1^, respectively. We found that this sequential doping process barely changed the molecular ordering of the polymer films but resulted in efficient molecular doping by forming a polymer-F4TCNQ ion pair. Thus, high organic thin-film transistor (OTFT) mobility is the key to increasing the electrical conductivity of OTE devices. Moreover, the π-extended backbones of PDTTz led to more efficient molecular doping and better charge carrier transport, resulting in three times higher electrical conductivity and PF than those of PDTz.

## 2. Materials and Methods

### 2.1. Materials

Anhydrous solvents, tri(o-tolyl)phosphine, tris(dibenzylideneacetone)dipalladium(0), trimethyltin chloride, and n-Butyllithium were purchased from Sigma-Aldrich, (Seoul, Republic of Korea). N-bromosuccinimide (NBS) was purchased from Alfa Aesar (Gongduk-Dong, Mapo-Gu, Republic of Korea) a, and thienothiophene, thiophene, and tetrakis(triphenylphosphine)-palladium(0) were purchased from TCI Chemical (Tokyo Chemical Industry Co., LTD., Tokyo, Japan). All chemicals were used without further purification. 4-(2-Ethylhexyl)-2,6-bis(trimethylstannyl)-4H-dithieno[3,2-b:2′,3′-d]pyrrole) were purchased from J’s science (Daejeon, Republic of Korea).

### 2.2. Synthesis of Monomers

2,5-Bis(4-octylthiazol-2-yl)thiophene (2): 2-Bromo-4-octylthiazole (1) (1.43 g, 6.1 mmol), 2,5-bis(trimethylstannyl)thiophene (1 g, 2.44 mmol), Pd_2_(dba)_3_ (65 mg, 0.07 mmol), and tri(o-tolyl)phosphine (74 mg, 0.24 mmol) were dissolved in N_2_-sparged toluene (40 mL). After the reaction mixture was refluxed at 110 °C for 48 h, it was cooled to RT and extracted with dichloromethane and water. The organic phase was dried over anhydrous MgSO_4_, and the solvent was removed by evaporation under reduced pressure. After solvent evaporation, the residue was purified using column chromatography on silica gel (methylene chloride (MC):hexanes = 2:3 *v*/*v*) to yield a brown oil (0.88 g, yield: 79%). ^1^H nuclear magnetic resonance (NMR) (400 MHz, CDCl_3_), δ (ppm): 7.46 (s, 2H), 6.85 (s, 2H), 2.80 (t, J = 8.0 Hz, 2H), 1.75 (m 2H), 1.35 (m, 10H), and 0.91 (t, J = 8.0 Hz, 3H).

2,5-Bis(5-bromo-4-octylthiazol-2-yl)thiophene (Tz): 2,5-Bis(4-octylthiazol-2-yl)thiophene (2) (1 g, 2.1 mmol) and N-bromosuccinimide (NBS) (0.94 g, 5.3 mmol) were dissolved in dimethylformamide (DMF) (60 mL) and stirred at RT overnight. The reaction mixture was poured into water. The organic phase was extracted with dichloromethane and water and dried over anhydrous MgSO_4_. The solvent was then removed by evaporation under reduced pressure. The resulting product was recrystallized using MC and methanol to yield a pure yellow solid (1.1 g, 83%). ^1^H NMR (400 MHz, CDCl_3_), δ (ppm): 7.20 (s, 2H), 2.66 (t, J = 8.0 Hz, 2H), 1.65 (m, 2H), 1.25 (m, 10H), and 0.82 (t, J = 8.0 Hz, 3H). ^13^C NMR (100 MHz, CDCl3), δ (ppm): 159.88, 157.22, 138.74, 126.64, 103.81, 32.02, 29.85, 29.55, 29.50, 29.35, 28.85, 22.83, and 14.28.

2,5-Bis(4-octylthiazol-2-yl)thieno[3,2-b]thiophene (3): 2-Bromo-4-octylthiazole (1) (1.43 g, 6.1 mmol), 2,5-bis(trimethylstannyl)thieno[3,2-b]thiophene (1.14 g, 2.44 mmol), Pd_2_(dba)_3_ (65 mg, 0.07 mmol), and tri(o-tolyl)phosphine (74 mg, 0.24 mmol) were dissolved in N_2_-sparged toluene (40 mL). After the reaction mixture was refluxed at 110 °C for 48 h, it was cooled to RT and extracted with dichloromethane and water. The organic phase was dried over anhydrous MgSO_4_, and the solvent was removed by evaporation under reduced pressure. After solvent evaporation, the resulting product was recrystallized using MC and methanol to yield a pure yellow solid (1.08 g, yield: 88%). ^1^H NMR (400 MHz, CDCl_3_), δ (ppm): 7.46 (s, 2H), 6.85 (s, 2H), 2.80 (t, J = 8.0 Hz, 2H), 1.75 (m 2H), 1.35 (m, 10H), and 0.91 (t, J = 8.0 Hz, 3H).

2,5-Bis(5-bromo-4-octylthiazol-2-yl)thieno[3,2-b]thiophene (TTz): 2,5-Bis(4-octylthiazol-2-yl)thieno[3,2-b]thiophene (3) (1 g, 1.88 mmol) and NBS (0.84 g, 4.7 mmol) were dissolved in DMF (60 mL) and stirred at RT overnight. The reaction mixture was then poured into water. The organic phase was extracted with dichloromethane and water and dried over anhydrous MgSO_4_. The solvent was then removed by evaporation under reduced pressure. After evaporating the solvent, the resulting product was recrystallized using MC and methanol to yield a pure yellow solid (1.18 g, 91%). ^1^H NMR (400 MHz, CDCl_3_), δ (ppm): 7.20 (s, 2H), 2.66 (t, J = 8.0 Hz, 2H), 1.65 (m, 2H), 1.25 (m, 10H), and 0.82 (t, J = 8.0 Hz, 3H). ^13^C NMR (100 MHz, CDCl3), δ (ppm): 159.88, 157.22, 138.74, 126.64, 103.81, 32.02, 29.85, 29.55, 29.50, 29.35, 28.85, 22.83, and 14.28.

### 2.3. Synthesis of Polymers

PDTz: 4-(2-Ethylhexyl)-2,6-bis(trimethylstannyl)-4H-dithieno[3,2-b:2′,3′-d]pyrrole (0.3 mmol), Tz (0.3 mmol), and a palladium catalyst, viz. Pd(PPh_3_)_4_ (0.009 mmol), were added to a 25 mL two-necked round-bottom flask under a nitrogen atmosphere. The mixture was dissolved in N_2_-sparged toluene (5 mL), stirred at 110 °C for 3 days, and then cooled to RT. The polymer was subsequently precipitated by adding methanol (200 mL) and filtering through a Soxhlet thimble. The precipitate was purified using Soxhlet extraction with acetone, hexane, and chloroform. The polymer was recovered as a solid from the chloroform fraction by precipitation with methanol. The solid was then dried under a vacuum (yield: 204 mg, 89%).

PDTTz: 4-(2-Ethylhexyl)-2,6-bis(trimethylstannyl)-4H-dithieno[3,2-b:2′,3′-d]pyrrole (0.3 mmol), TTz (0.3 mmol), and a palladium catalyst, Pd(PPh_3_)_4_ (0.009 mmol) were added to a 25 mL two-necked round-bottom flask under a nitrogen atmosphere. The mixture was dissolved in N_2_-sparged toluene (5 mL), stirred at 110 °C for 3 days, and then cooled to RT. The polymer was subsequently precipitated by adding methanol (200 mL) and filtering through a Soxhlet thimble. The precipitate was purified using Soxhlet extraction with acetone, hexane, and chloroform. The polymer was recovered as a solid from the chloroform fraction by precipitation with methanol. The solid was then dried under a vacuum (yield: 226 g, 92%).

### 2.4. Characterization of the Materials

Tetrafluoro-tetracyanoquinodimethane (F4TCNQ) (98%), o-1,2-dichlorobenzene (DCB; anhydrous, 99%), acetonitrile (ACN; anhydrous, 99.8%), and chloroform (anhydrous, 99%) were purchased from Sigma–Aldrich (Seoul, Korea). All chemicals were used as received without purification. The ^1^H NMR spectra were recorded at 25 °C on a VNMRS 600 MHz spectrometer, with tetramethylsilane as an internal reference in CDCl_3_. The UV–vis–NIR spectra were measured using a spectrophotometer (V670, JACSO). The Raman spectra were measured using a Raman spectrometer (InVia Raman Microscope, Renishaw, Wotton-under-Edge, United Kingdom) at an excitation wavelength of 785 nm. Cyclic voltammetry was performed at a scan rate of 30 mV s ^−1^ on a WonATech potentiostat/galvanostat/impedance analyzer ZIVE SP1(1A) (Seoul, Republic of Korea) with a three-electrode cell with 0.1 N Bu_4_NBF_4_ solution in acetonitrile. The polymer film was coated onto the working electrode by dipping it in a polymer solution in chloroform. All measurements were calibrated against an internal ferrocene (Fc) standard, the ionization potential (IP) of which is −4.8 eV for the Fc/Fc^+^ redox system. Gel permeation chromatography (GPC) measurements were conducted at 35 °C using an Agilent 1260 Infinity II (California, USA). Gaussian 16 was used for electronic structure modeling of the polymers [49]. Density functional theory (DFT) calculation was performed using the B3LYP method with a 6-31G basis set. The input command was # opt b3lyp/6-31g (d,p) geom = connectivity, charge = 0, and multiplicity = 1 [50,51].

### 2.5. Two-Dimensional Grazing-Incidence X-ray Diffraction (2D-GIXD) Experiments

The 2D-GIXD measurements were performed under vacuum at the PLS-II 9A beamline of the Pohang Accelerator Laboratory in Korea. The samples were prepared on Si wafers covered with thermally grown 100-nm-thick SiO_2_ dielectric layers. The beam energy of the X-ray was 10.08 keV, and the incidence angle was 0.16°. The 2D-GIXD images from the films were analyzed based on the relationship between the scattering vector (*q*) and the d-spacing (*q* = 2*π/d*).

### 2.6. Sample Preparation

Soda-lime glass (2 × 2 cm) was cleaned by bath sonication in diluted Hellmanex III (2 vol%), acetone, and ethanol in sequence. To prepare sequentially doped films, PDTz and PDTTz polymers (10 mg mL^−1^) and F4TCNQ (0.5–2.0 mg mL^−1^) were dissolved in DCB and ACN, respectively. The PDTz and PDTTz solutions were spin-coated onto cleaned glass substrates at 1000 rpm for 60 s. The obtained PDTz and PDTTz films were annealed on a 150 °C hot plate for 10 min. The annealed films were rotated on a spin coater at 3000 rpm, and the F4TCNQ solution (100 µL) was poured onto the films. Heavily n-doped Si wafers covered with thermally grown 100 nm-thick SiO_2_ dielectric layers were used as substrates for the 2D-GIXD measurements.

For OTFT device fabrication with a top-contact/bottom-gate configuration, heavily n-doped Si wafers covered with thermally grown 300 nm-thick SiO_2_ dielectric layers were used as the gate substrates. The wafers were cleaned by piranha treatment, repeatedly rinsed, and sonicated in a deionized water bath. The as-cleaned wafers were then treated with a UV-ozone cleaner for 20 min. The wafers were immersed in an octadecyltrichlorosilane (ODTS) solution (1 vol% in anhydrous toluene) for 1 h in ambient air to remove polar hydroxyl groups on the wafer surface [52]. The PDTz and PDTTz solutions (5 mg mL^−1^ in chloroform) were spin-coated at 2000 rpm for 45 s on the ODTS-treated wafers. The polymer films were annealed for 10 min on a hot plate at various temperatures and then slowly cooled to RT. All device fabrication processes were performed in an N_2_-filled glove box (O_2_ and H_2_O < 5 ppm). Next, a 100 nm-thick Au source and drain electrodes were thermally deposited onto the active layers with a shadow mask via a thermal evaporator; the channel length (*L*) and width (*W*) were 100 and 300 µm, respectively.

### 2.7. Device Characterization

The TE properties of the doped PDTz and PDTTz films were measured in an N_2_-filled glove box. Sheet resistance was measured using a 4-point probe (each tip was 1 mm apart) connected to a Keithley 2400 source meter. The film thicknesses were measured using a surface profiler (Alpha Step IQ, KLA Tencor, Milpitas, CA, USA). The electrical conductivity was calculated from the measured sheet resistance and thickness. The Seebeck coefficient was measured using a custom-built setup used in previous studies [53,54,55]. To apply the temperature differences, two Peltier devices were operated using a Keithley 2400 source meter in a dependent manner. The temperature was measured using a Keithley 2700 instrument (Cleveland, OH, USA). The voltage was measured using a Keithley 2182A nanovoltmeter at five different temperature differences of <2 K at an average of 300 K. The Seebeck coefficient was extracted from the slope of the voltage versus temperature difference plot.

The transfer and output curves of the OTFTs were measured using a Keithley 4200-SCS parameter analyzer in an N_2_-filled glove box. The field-effect mobility was extracted from the transfer curve in the saturation regime measured at *V*_D_ = −80 V as follows: *I*_D_ = *μC*_i_(*W*/2*L*)(*V*_G_ − *V*_th_)^2^, where *V*_D_ is the drain voltage, *I*_D_ is the drain current, *C*_i_ is the capacitance per unit area (10.0 nF cm^−^^2^), *V*_G_ is the gate voltage, and *V*_th_ is the threshold voltage.

## 3. Results and Discussion

### 3.1. Synthesis and Characterization of Materials

2-Bromo-4-octylthiazole (1), 2,5-bis(trimethylstannyl)thiophene, and 2,5-bis(trimethylstannyl)thieno[3,2-b]thiophene were synthesized according to procedures mentioned in the literature [48]. In particular, our synthetic process for compounds 1, 2, and 3 did not require any column chromatography purification of the starting materials, which is advantageous for the mass production of monomers. Two monomers, Tz and TTz, were obtained by Stille coupling of compound 1 with compounds 2 and 3, respectively, followed by bromination with NBS. Both monomers (Tz and TTz) were obtained with total yields of 48 and 58% from the starting materials.

The final TE polymers, PDTz and PDTTz, were synthesized via Stille polycondensation of the DTP-based monomer with Tz and TTz comonomers, respectively. The synthesis procedure is shown in Scheme 1. The synthesized polymers were purified via Soxhlet extraction using methanol, acetone, hexanes, and chloroform. The average molecular weight (*M*_w_) and dispersity (*Đ = M_w_/M*_n_) of the polymers were measured using GPC against polystyrene with chloroform as the eluent. The *M*_w_ of PDTz and PDTTz were 13.6 and 13.8 kDa, respectively, and their *Đ* were 1.23 and 1.17, respectively (Figures S8 and S9). From Soxhlet extraction, the narrow *Đ* and similar molecular weight of the polymers could be obtained, which was crucial for comparing the OTFT and OTE performance of the two polymers. The synthesized monomers and polymers were identified by NMR spectroscopy (Figures S1–S7).

### 3.2. Optical and Electrochemical Properties

**Figure 1:**
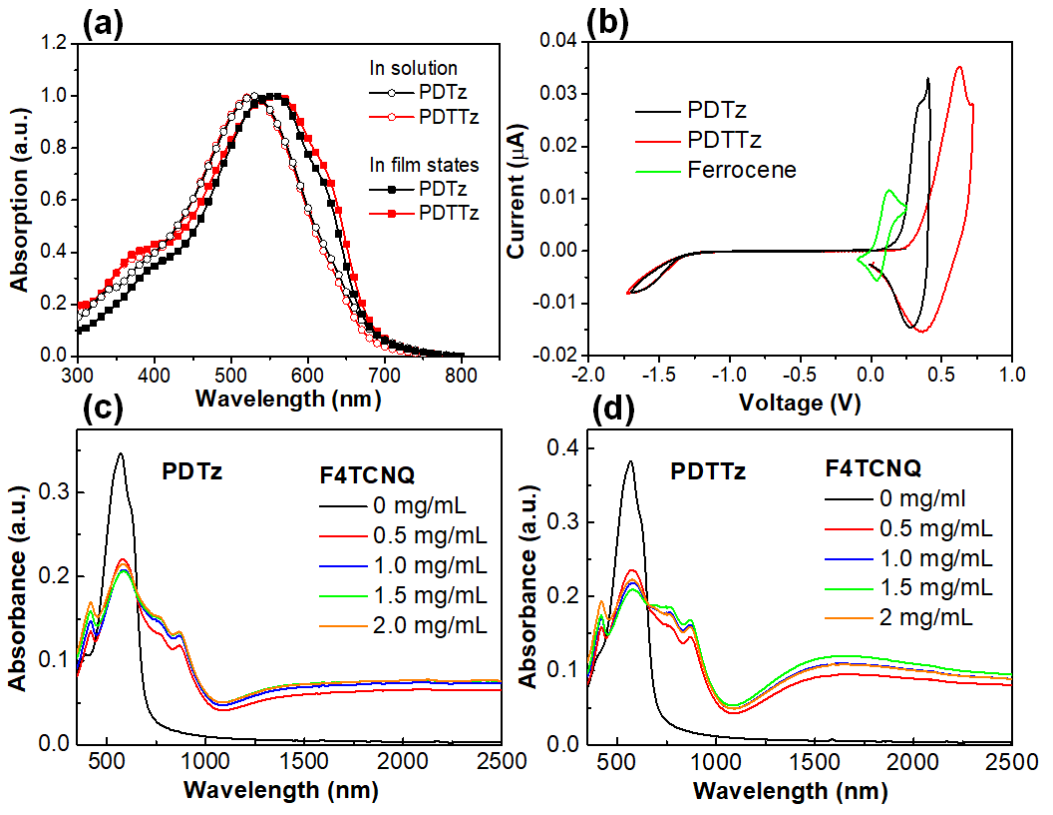
(**a**) Absorption spectra of PDTz and PDTTz in solution and film states. (**b**) Cyclic voltammogram of the polymer films. Absorption spectra of doped (**c**) PDTz and (**d**) PDTTz polymers depending on F4TCNQ concentration (0.5–2.0 mg mL^−^^1^).

The UV-vis-NIR absorption spectra of PDTz and PDTTz were measured in the solution and film states (Figure 1a). Interestingly, PDTz and PDTTz showed similar absorption profiles for both solution and film. This indicates that both polymers engage in strong π-π intermolecular interactions, even in a diluted solution, by regular adjacent chain folds. The maximum absorption peaks (λ_max_) of PDTz and PDTTz were 528 nm for both in the solution state and 554 nm and 557 nm, respectively, in the film states, which corresponds to *π*-π* transition. In the film states, the λ_max_ of PDTTz was red-shifted, and shoulder absorption was more clearly observed than that of PDTz. This indicates that the PDTTz polymer has better π-π stacking and molecular ordering than the PDTz polymer. The optical bandgaps ($E_{g}^{\;opt}$) of PDTz and PDTTz were calculated to be 1.81 and 1.79 eV, respectively, from the absorption onset wavelength in the film state (Table 1).

Cyclic voltammetry (CV) was measured to determine the highest occupied molecular orbital (HOMO) energy levels of the synthesized polymers (Figure 1b and Table 1). The oxidation onset potentials of PDTz and PDTTz were 0.16 and 0.27 V, respectively, and their corresponding HOMO energy levels were −4.9 and −5.0 eV, respectively. The HOMO energy levels of both polymers were shallow enough for efficient ground-state charge transfer, with the F4TCNQ dopant having a lowest unoccupied molecular orbital (LUMO) energy level of −5.24 eV [56].

Figure 1c,d show the UV-vis-NIR absorption spectra of doped PDTz and PDTTz polymers depending on F4TCNQ concentration (0.5–2.0 mg mL^−^^1^) using sequential processing. Adding F4TCNQ to the polymer films resulted in new absorption peaks in the range of 750–1000 nm and above 1087 nm. These absorption peaks indicate that the PDTz and PDTTz polaron states were formed by successful molecular doping with F4TCNQ. As the concentration of F4TCNQ increased, the *π*-π* transition peak from the neutral polymers decreased, but the polaron absorption peaks increased and were almost saturated at an F4TCNQ concentration of 1.5 mg mL^−^^1^. At higher concentrations, there was no difference in the doping. This means that sequential doping of the polymer films is quite efficient, and no more than 1.5 mg mL^−^^1^ of F4TCNQ is required to optimize the OTE devices.

As shown in Figure 1c,d, PDTTz showed much stronger polaronic absorption peaks above 1085 nm in the doped states than PDTz, indicating that PDTTZ has better doping efficiency. We believe that this doping efficiency is related to the larger backbone structure of PDTTz, which provides more space for inserting the ionized F4TCNQ dopant [57].

### 3.3. Thermoelectric Properties

**Figure 2:**
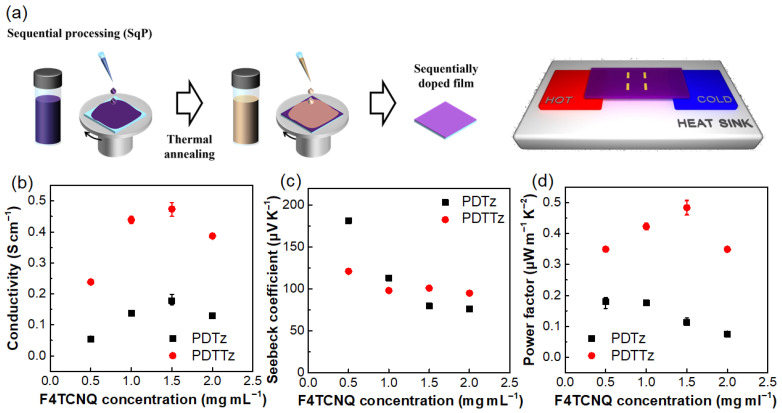
(**a**) Layer structure of OTE devices. (**b**) Seebeck coefficient, (**c**) conductivity, and (**d**) power factor of PDTz and PDTTz devices.

The thermoelectric performances of PDTz and PDTTz were measured by increasing the concentration of the F4TCNQ dopant. The device fabrication process is shown in Figure 2a. As the dopant concentration increased, the electrical conductivities of PDTz and PDTTz gradually increased, mainly because of the increased charge carrier density. The maximum electrical conductivities of PDTz and PDTTz were determined to be 0.18 ± 0.02 and 0.48 ± 0.02 S cm^−^^1^, respectively, at a dopant concentration of 1.5 mg mL^−^^1^ (Figure 2b). However, at concentrations higher than 1.5 mg mL^−^^1^, doping rarely occurred, as shown in Figure 2b,c. This indicates that the extra dopant acted as an impurity, preventing efficient molecular doping and charge transport in the OTE devices.

**Figure 3:**
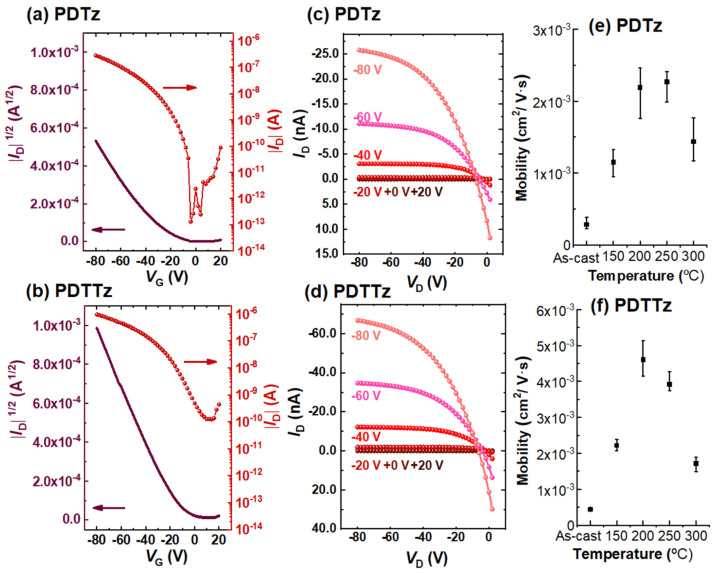
OTFT characteristics. Transfer curve of (**a**) PDTz and (**b**) PDTTz displaying the saturation regime for V_g_ = −80 V, and output curves of (**c**) PDTz and (**d**) PDTTz taken at different gate voltages. Charge carrier mobility of (**e**) PDTz and (**f**) PDTTz at different temperatures.

In particular, the electrical conductivity of PDTTz was almost three times higher than that of PDTz at the same dopant concentration. Because electrical conductivity is proportional to the product of the number of charge carriers and mobility, higher electrical conductivity can result from higher hole mobility of the polymer. As shown in Figure 3, the OTFT devices were fabricated without F4TCNQ doping to compare the mobilities of the two polymers. The hole mobility of the two polymers was maximized via thermal annealing at 200–250 °C and then decreased at higher temperatures due to thermal decomposition [58]. The average hole mobilities of PDTz and PDTTz were 2.2 × 10^−^^3^ and 4.6 × 10^−^^3^ cm^2^ V^−^^1^ s^−^^1^, respectively, in the saturation regime at *V*_g_ of −80 V (Figure 3a,b). The hole mobility of PDTTz was twice that of PDTz, which is crucial for the higher electrical conductivity of PDTTz in OTE devices.

The Seebeck coefficient has a trade-off relationship with electrical conductivity. Thus, a higher polymer doping level led to a more significant decrease in the Seebeck coefficient. As shown in Figure 2c, the Seebeck coefficient decreased as the F4TCNQ concentration increased but became constant when the dopant concentration reached 1.5 mg mL^−^^1^. This indicates that molecular doping effectively occurred up to a dopant concentration of 1.5 mg mL^−^^1^, but no further doping occurred at a dopant concentration above 1.5 mg mL^−^^1^. This agrees with the change in the absorption spectrum that occurs with increasing dopant concentrations (Figure 1c,d).

The PF (S^2^σ) indicates the electrical properties of TE materials. The highest PFs of the PDTz and PDTTz devices were 0.18 ± 0.01 µW m^−^^1^ K^−^^2^ at an F4TCNQ concentration of 0.5 mg mL^−^^1^ and 0.48 ± 0.02 µW m^−^^1^ K^−^^2^ at an F4TCNQ concentration of 1.5 mg mL^−^^1^, respectively (Figure 2d and Table 2). PDTTz devices showed three times higher PFs than PDTz devices. The superior doping efficiency and higher hole mobility of PDTTz could result in better OTE performance.

**Figure 4:**
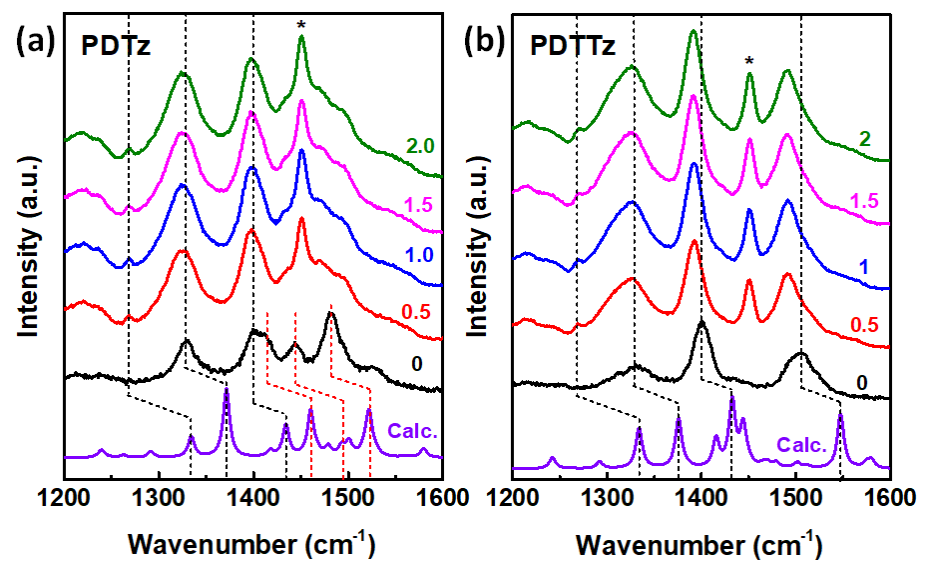
Raman spectra of (**a**) PDTz and (**b**) PDTTz films depending on the F4TCNQ doping ratio. Asterisks denote a new transition that increases with F4TCNQ doping ratio.

Raman spectroscopy was performed to investigate the conformational structures of the pristine and doped PDTz and PDTTz films (Figure 4). The conformational structures of doped polymers are correlated with their intramolecular charge transport properties [59]. A laser wavelength of 785 nm was chosen to consider the charge transport in the doped polymer (i.e., the polaron state). In addition, the stretching modes of PDTz and PDTTz were simulated from DFT calculation using the B3LYP method with a 6-31G (d,p) basis set [49] to understand the origin of each Raman band in the experimental data (Figure 4, Figures S10 and S11) [60,61]. As shown in Figure 4, the Raman spectra of the polymers showed several bands at 1200–1600 cm^–1^, with some red-shifted after doping. The DTP moiety’s C-N-C stretching mode at 1400 cm^–1^ was representatively red-shifted to 1397 cm^–1^ for PDTz and 1391 cm^–1^ for PDTTz. The degree of the red shift means that PDTTz has more π-extended polymer backbones after doping than PDTz. These results agree with the higher electrical conductivities and PFs of PDTTz compared to those of PDTz.

### 3.4. Morphological Properties

**Figure 5:**
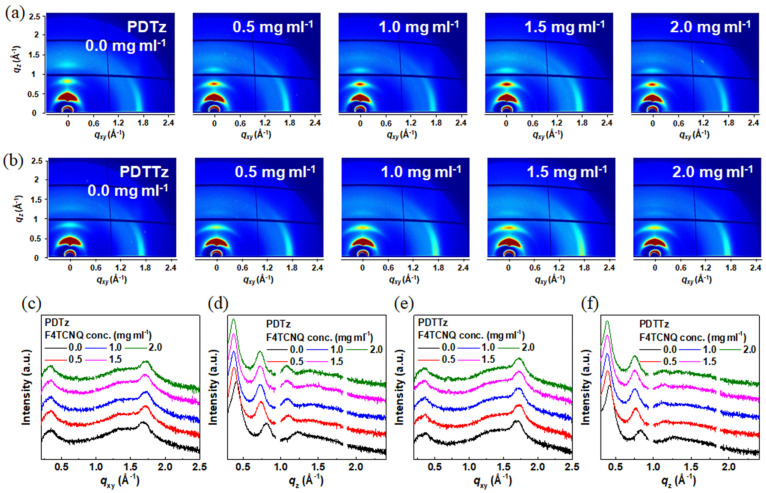
2D-GIXD data of (**a**) PDTz and (**b**) PDTTz films depending on the F4TCNQ doping ratio. Corresponding line-cut spectra of (**c**,**d**) PDTz and (**e**,**f**) PDTTz films along the (**c**,**e**) in-plane (*q*_xy_) and (**d**,**f**) out-of-plane (*q*_z_) directions.

2D-GIXD was carried out to investigate the correlation between the microstructure of the polymer film and TE performance (Figure 5a,b). PDTz and PDTTz exhibited clear (*h*00) peaks (*h* = 1, 2, and 3) along the out-of-plane direction (*q_z_*), with d-spacings of 15.14 and 14.84 Å, respectively (Figure 5d,f). In addition, the (010) π-π stacking peaks of PDTz and PDTTz were also clearly observed with d-spacings of 3.71 and 3.69 Å, respectively, along the in-plane direction (*q_xy_*) (Figure 5c,e). Both polymers exhibited a dominant edge-on orientation, which is beneficial for charge carrier transport in OTE devices. In comparing the two doped polymers, doped PDTTz had a slightly less ordered but closely packed structure than doped PDTz.

Notably, the doping process barely changed the molecular ordering and orientation of the polymers. As shown in Figure 5a,b, the lamellar ordering and edge-on orientation were similar before and after doping. However, the d-spacings of both polymers (100) and (010) peaks gradually increased and decreased depending on the dopant concentration. This indicates that F4TCNQ was inserted into the polymer lamellar structure without disturbing the alignment of the polymer (Figure 5c–f and Table S1) [62]. When comparing the two polymers, the d-spacings of the (100) peak PDTz and PDTTz were 16.55 and 16.06 Å, respectively, after doping. The smaller d-spacing of the doped PDTTz films was more favorable for transporting free charge carriers in OTE devices.

**Figure 6:**
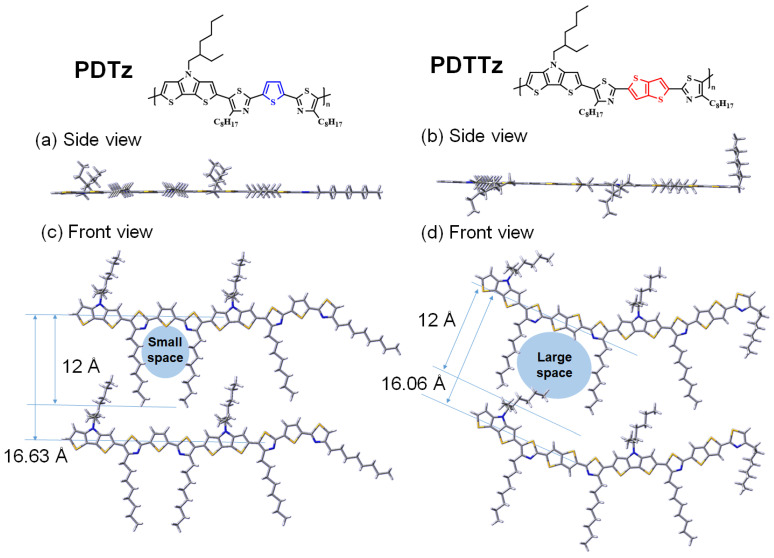
Optimization of the dimer structure of polymer repeat units via DFT calculation. Side view of the optimized (**a**) PDTz and (**b**) PDTTz structures. Estimated space within the lamellar structure of (**c**) PDTz and (**d**) PDTTz.

The two repeating units of the PDTz and PDTTz polymer backbones were simulated from DFT calculations using the B3LYP method with a 6-31G (d,p) basis set [49]. As shown in Figure 6a,b, both polymer backbones have a highly planar backbone structure with dihedral angles lower than 5°. The newly synthesized alkylthiazole-based Tz and TTz monomers were highly efficient in increasing the backbone planarity of the polymers, owing to the dipole-dipole interaction between the nitrogen of 4-octylthiazole and the adjacent thiophene (or fused thiophene) ring. As a result, both polymers can form efficient intra- and intermolecular interactions in the film state, which facilitate charge transport in the devices.

Moreover, as shown in Figure 6c,d, we estimated the empty space in the lamellar structures of the polymers. There was substantial intercalation of the alkyl side chains because the d-spacing of the doped lamellar structure was ~16 Å, but the length of the alkyl side chains was ~12 Å. Thus, more space between the alkyl side chains provides more room for inserting the F4TCNQ dopant into the lamellar structure. Comparing the two polymers, PDTTz containing TTz monomers can create more space because of the larger size of thienothiophene. Thus, the use of PDTTz could result in better doping efficiency in the film state [57]. Introducing a TTz monomer in a polymer backbone has a dual effect of forming a closely packed molecular structure and improving the doping efficiency.

## 4. Conclusions

We synthesized two new alkylthiazole-based comonomers, Tz and TTz, and prepared the conjugated copolymers PDTz and PDTTz by Stille coupling of Tz and TTz, respectively, with a DTP comonomer. The DTP moiety provided strong electron-donating properties to the polymer, which offered excellent doping properties with the F4TCNQ organic dopant via sequential doping. Notably, the Tz and TTz monomers significantly improved the backbone planarity of the polymers and led to strong molecular ordering and edge-on orientation of the polymers in the film state, even in the presence of the F4TCNQ organic dopant. When comparing pristine PDTz and PDTTz films, PDTTz containing a thienothiophene moiety showed stronger ordering and more π-extended backbone, resulting in high hole mobility. This high hole moiety in the OTFTs was key to increasing the electrical conductivity of the OTE devices fabricated using the sequential doping method because the process barely changed the molecular ordering of the polymer films. Moreover, PDTTz showed better doping efficiency than PDTz because the PDTTz-containing TTz monomers could create more space in the lamellar structure for the ionized F4TCNQ dopant. As a result, PDTTz showed three times higher electrical conductivity of 0.47 ± 0.02 S cm^−^^1^ and PF of 0.48 ± 0.02 µW m^−^^1^ K^−^^2^ than PDTz.

## Figures and Tables

**Table 1:** Optical and electrochemical properties of polymers.

	λ_max_ (nm) ^a^		*E*_g_^opt^ (eV) ^b^	*E*_ox_ (V)/*E*_HOMO_ (eV) ^c^	*E*_LUMO_ (eV) ^d^
	Solution ^a^	Film			
PDTz	528	554	1.81	0.16/−4.9	−3.09
PDTTz	528	557	1.79	0.27/−5.0	−3.21

^a^ Maximum absorption wavelength; ^b^ optical bandgap; ^c^ HOMO energy level from CV; ^d^ LUMO energy level from *E*_HOMO_ and *E*_g_^opt^.

**Table 2:** Summary of OTFT and OTE properties.

Polymer	Mobility (cm^2^ V^−1^ s^−1^)	Electrical Conductivity σ (S cm^−1^)	Seebeck Coefficient S (µV K^−1^)	Power Factor S^2^σ (µW m^−1^ K^−2^)
PDTz	2.2 × 10^−3^	0.05 ± 0.004	181	0.18 ± 0.01
PDTTz	4.6 × 10^−3^	0.47 ± 0.02	101	0.48 ± 0.02

## Data Availability

Data are available upon request.

## Supplementary Materials

The following supporting information can be downloaded at: https://www.mdpi.com/article/10.3390/nano13071286/s1, Figure S1: ^1^H NMR of compound **1**; Figure S2: ^1^H NMR of compound **2**; Figure S3: ^1^H NMR of compound **3**; Figure S4: ^1^H NMR of compound **Tz**; Figure S5: ^1^H NMR of compound **TTz**; Figure S6: ^1^H NMR of PDTz; Figure S7: ^1^H NMR of PDTTz; Figure S8: GPC spectrum of PDTz; Figure S9: GPC spectrum of PDTTz; Figure S10: DFT-calculated stretching modes of the PDTz tetramer. The wavenumbers in parentheses indicate experimental values; Figure S11: DFT-calculated stretching modes of the PDTTz tetramer. The wavenumbers in parentheses indicate experimental values; Table S1: d-spacing of (100) and (010) peaks in Figure 5c–f.
