# Supplementary material for: Development of Alkylthiazole-Based Novel Thermoelectric Conjugated Polymers for Facile Organic Doping
Source: Nanomaterials (Basel). 2023 Apr 6;13(7):1286. doi: 10.3390/nano13071286 (PMC10097314; doi:10.3390/nano13071286)
Supplement: Supplementary file 1 [file nanomaterials-13-01286-s001.zip › nanomaterials-2277649-supplementary.pdf]

Supporting Information

Development of Alkylthiazole-Based Novel Thermoelectric Conjugated Polymers for Facile Organic Doping

Junho Kim [1], Eui Hyun Suh [1], Kyumin Lee [1], Gyuri Kim [2], Hansu Kim [1], Jaeyoung Jang [1,*] and In Hwan Jung [2,*]

[1] Department of Energy Engineering, Hanyang University, Seoul 04763, Republic of Korea
[2] Department of Organic and Nano Engineering, and Human-Tech Convergence Program, Hanyang University, 222 Wangsimni-ro, Seongdong-gu, Seoul 04763, Republic of Korea
[*] Correspondence: jyjang15@hanyang.ac.kr (J.J.); inhjung@hanyang.ac.kr (I.H.J.)

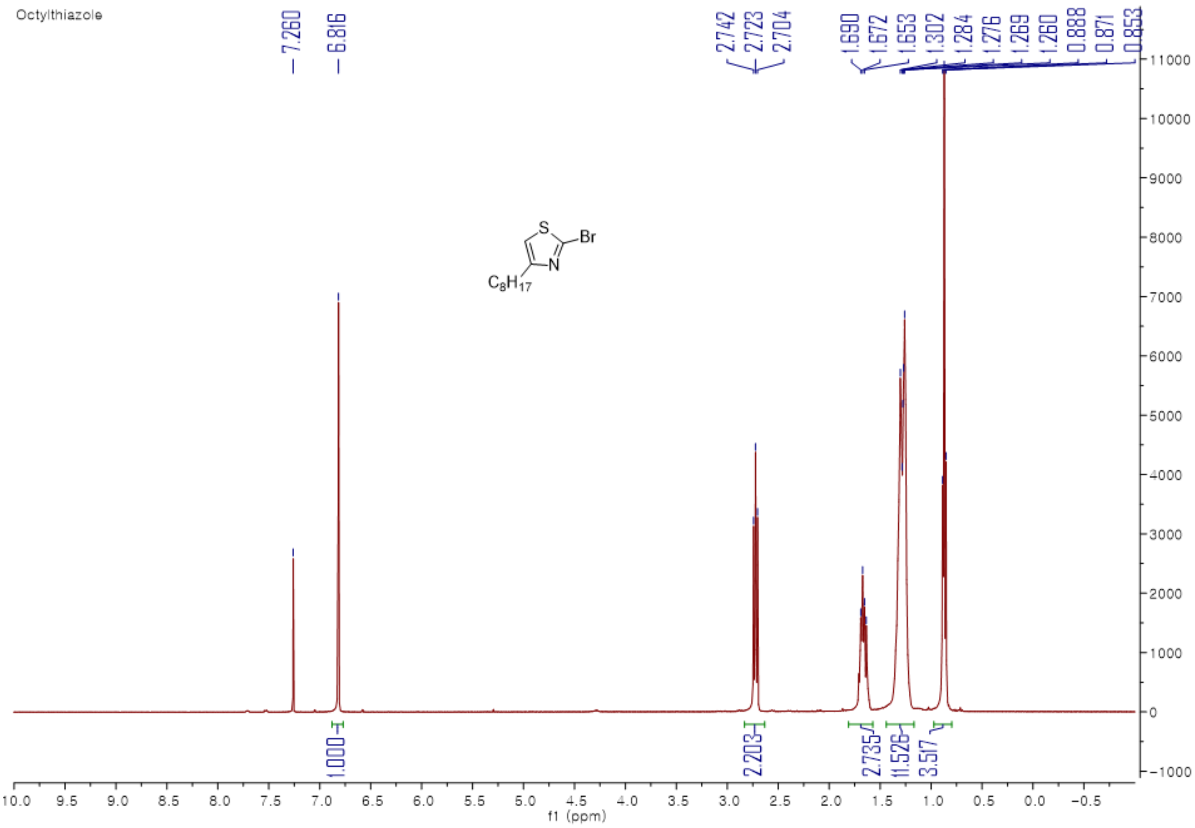

Figure S1. ^{1}H NMR spectrum of compound 1

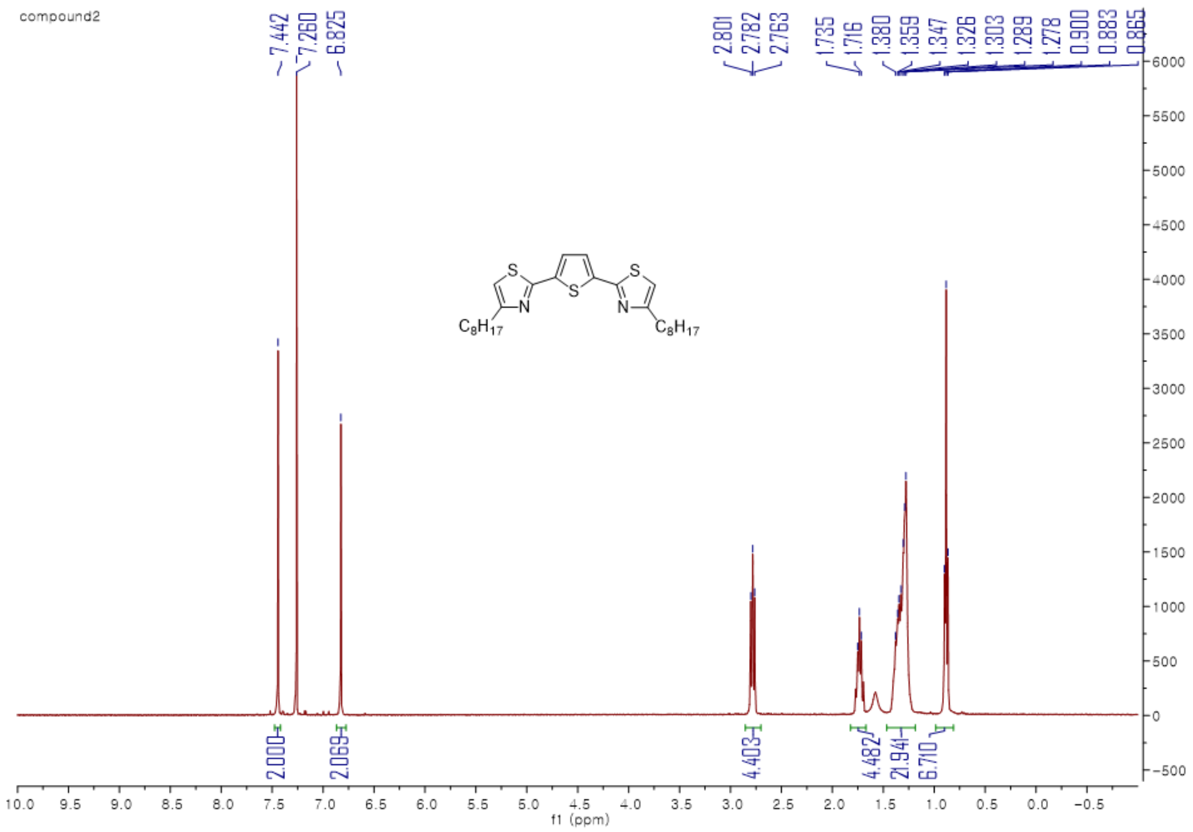

Figure S2. ^{1}H NMR spectrum of compound 2

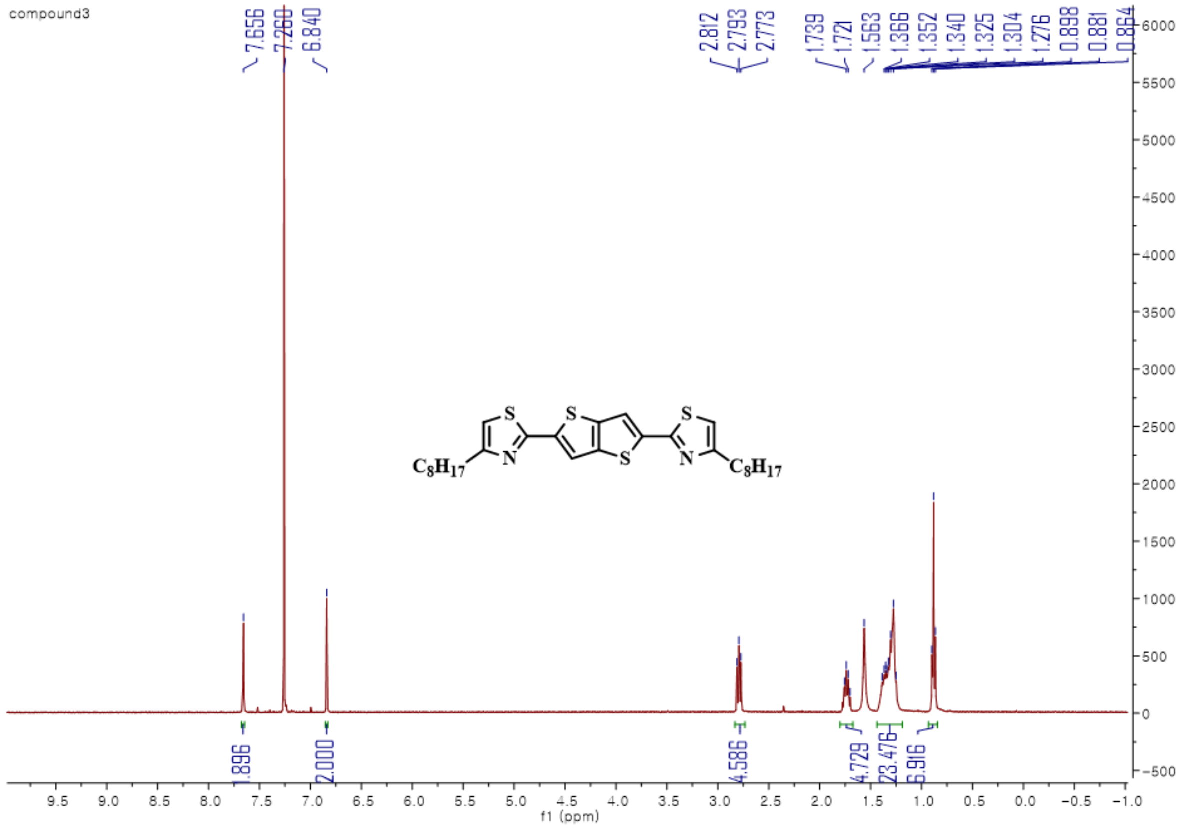

Figure S3. ^{1}H NMR of compound 3

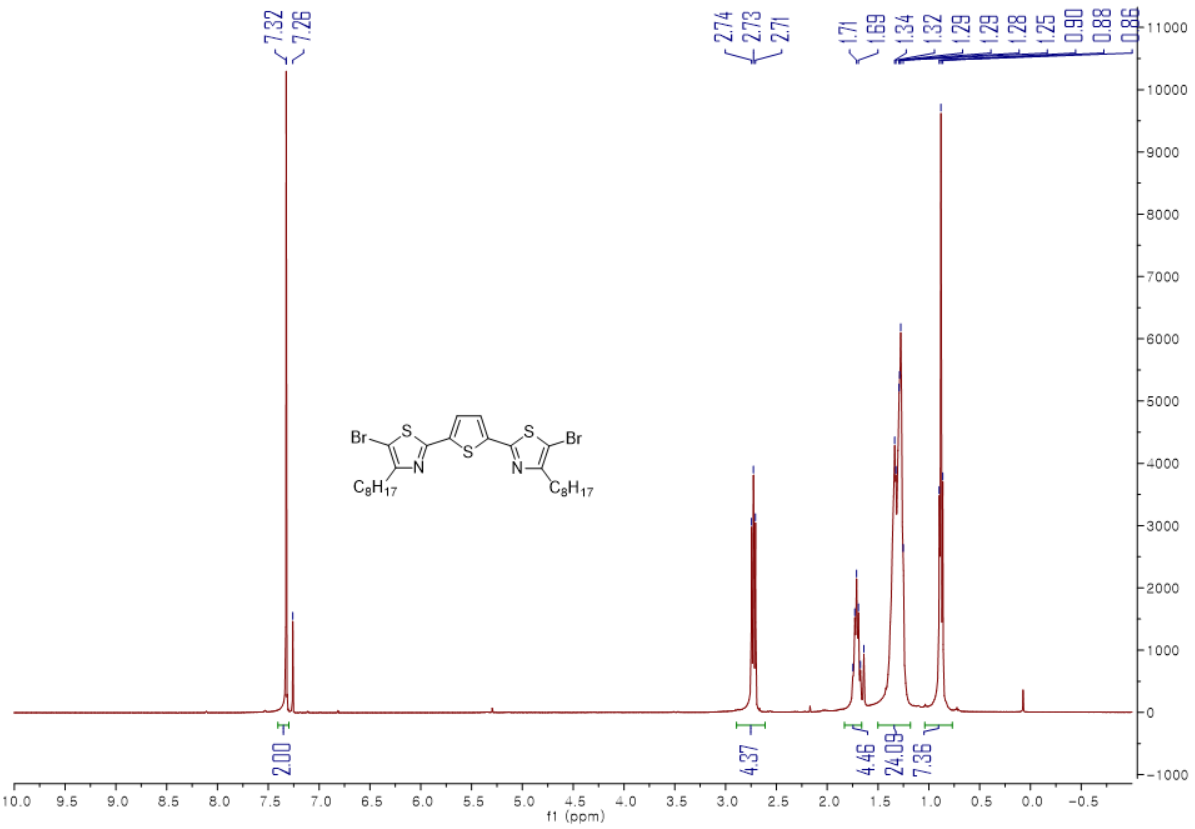

Figure S4. ^{1}H NMR spectrum of Tz

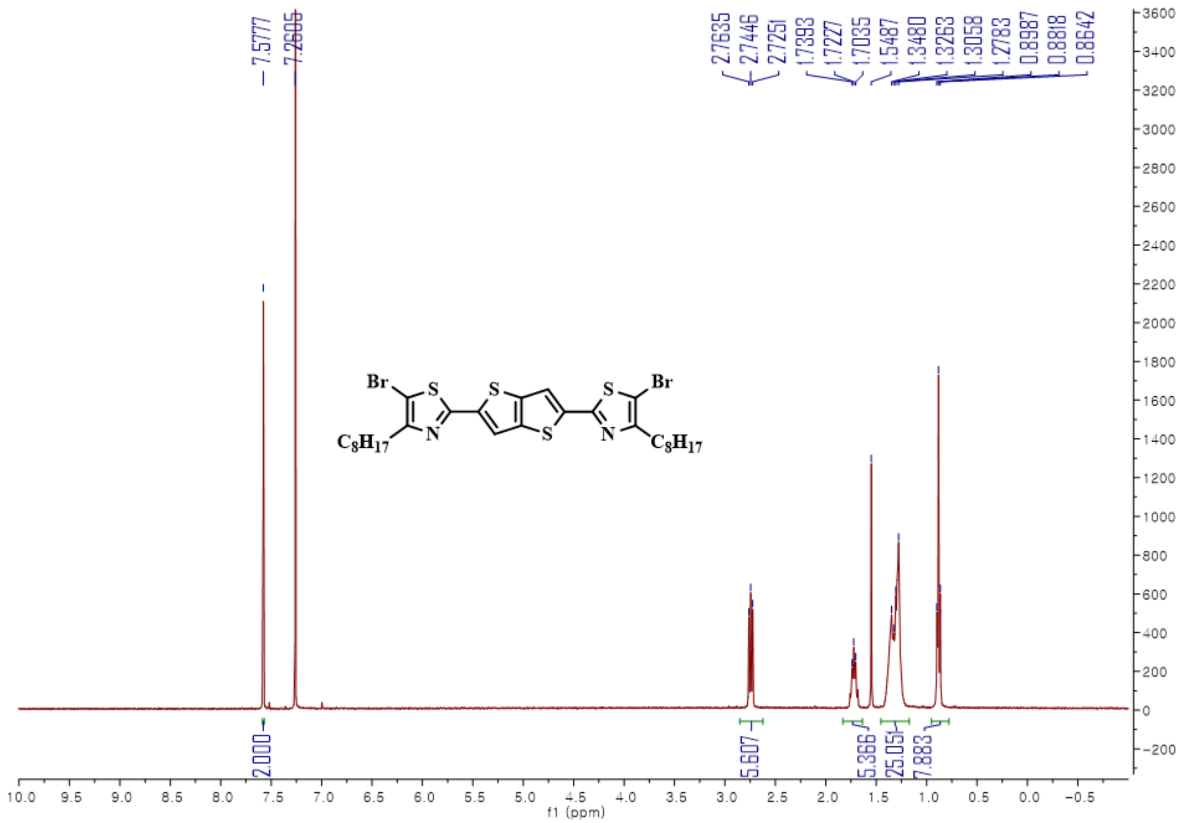

Figure S5. ^{1}H NMR spectrum of TTz

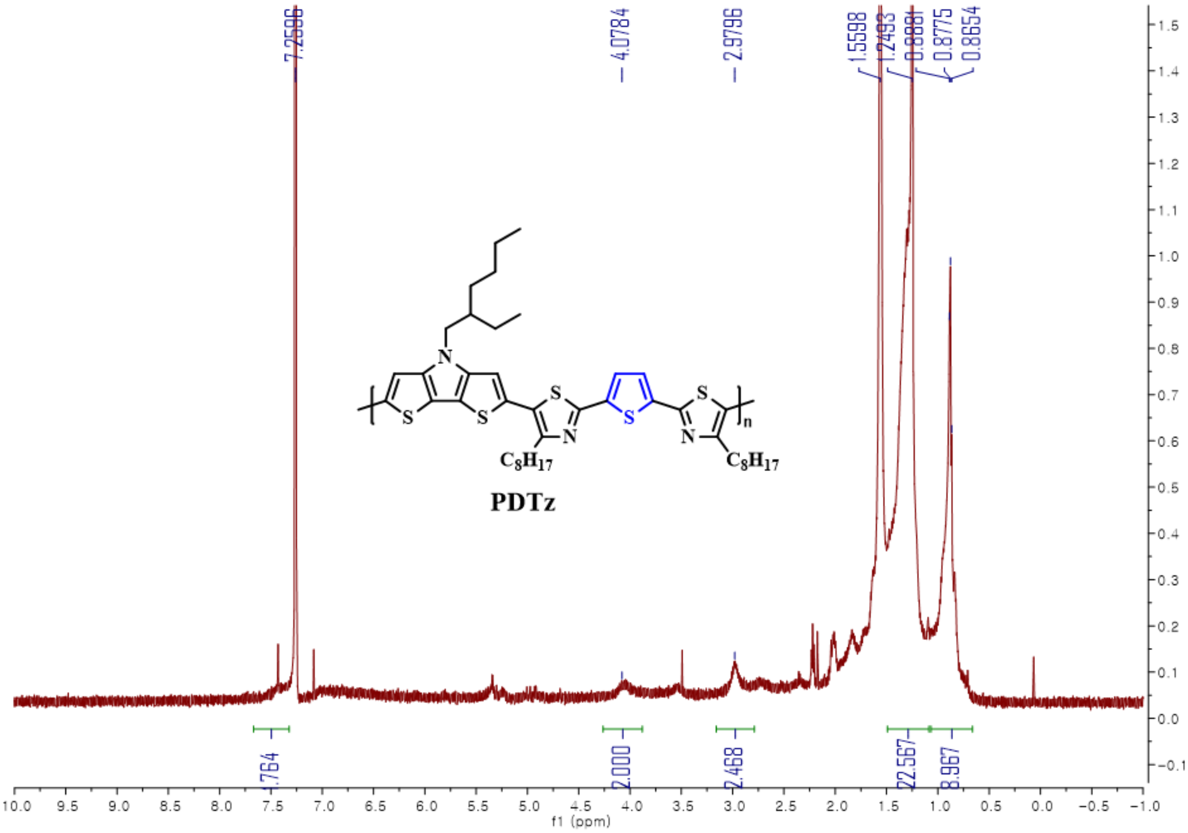

Figure S6. ^{1}H NMR spectrum of PDTz

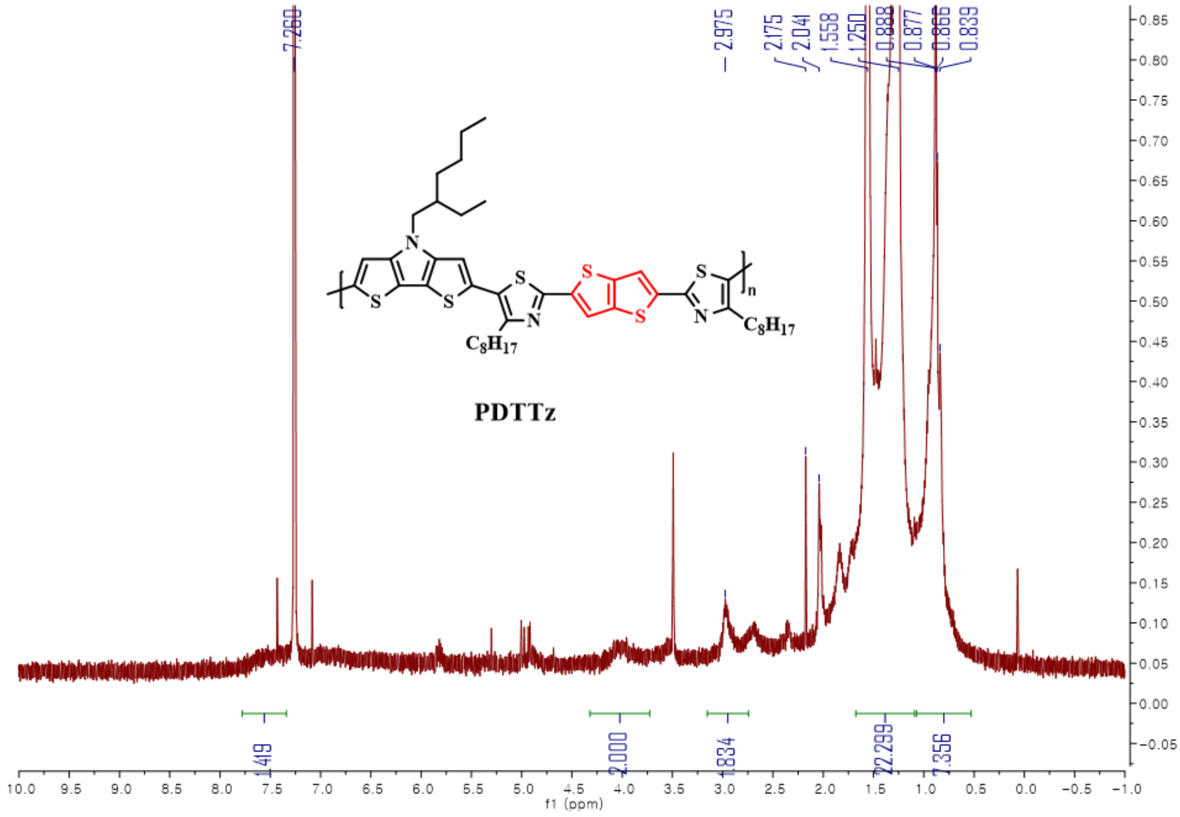

Figure S7. ^{1}H NMR spectrum of PDTTz

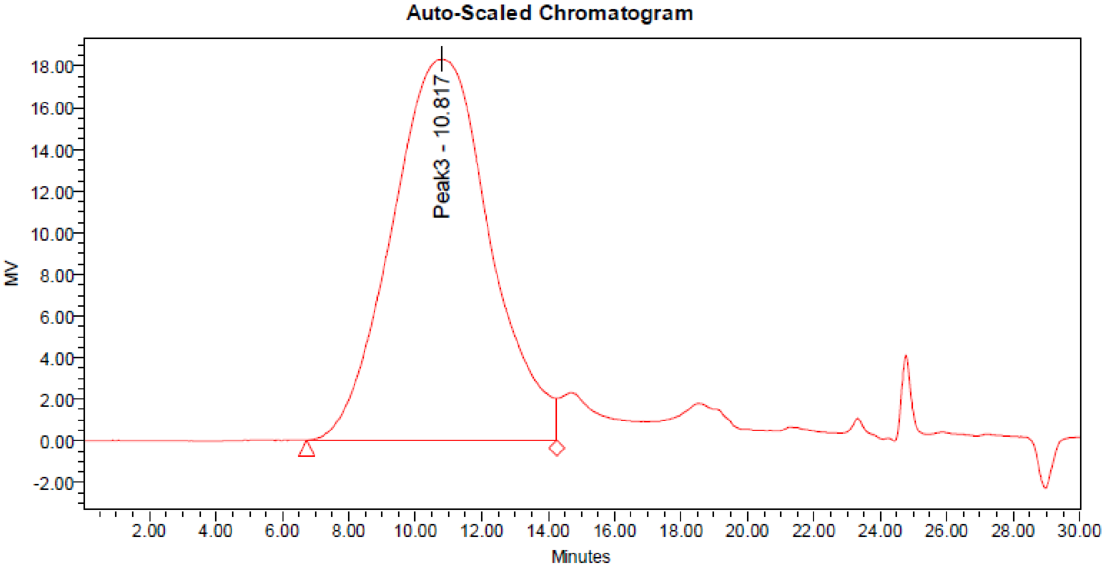

GPC Results

	Dist Name	Mn	Mw	MP	Mz	Mz+1	Mv	Polydispersity	MW Marker 1	MW Marker 2
1		110732	136620		159692	176983		1.233790		
2										

Figure S8. GPC spectrum of PDTz

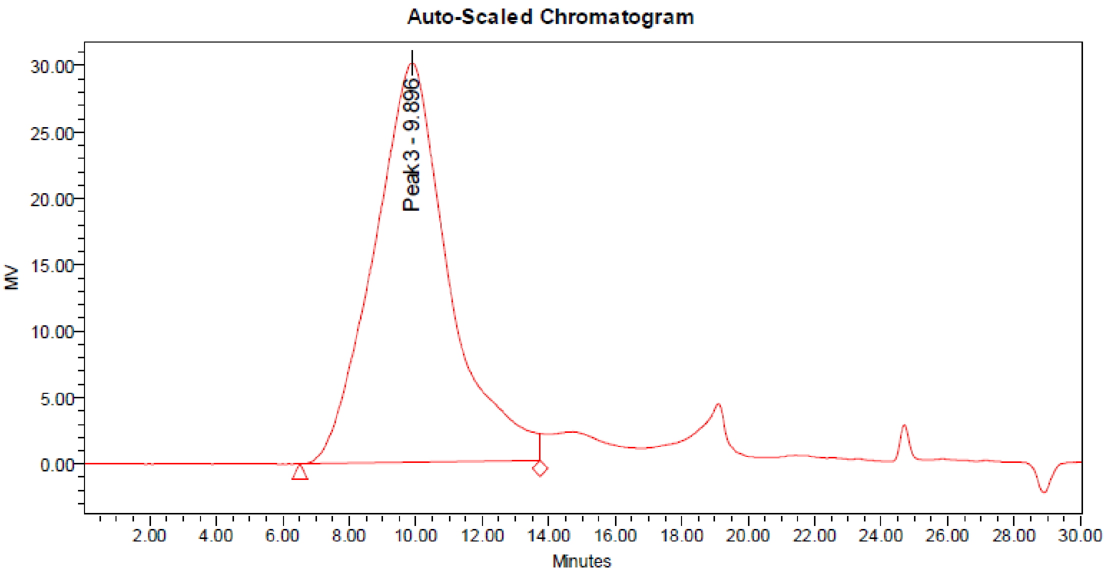

GPC Results

	Dist Name	Mn	Mw	MP	Mz	Mz+1	Mv	Polydispersity	MW Marker 1	MW Marker 2
1		118993	138814		158085	174140		1.166578		
2										

Figure S9. GPC spectrum of PDTTz

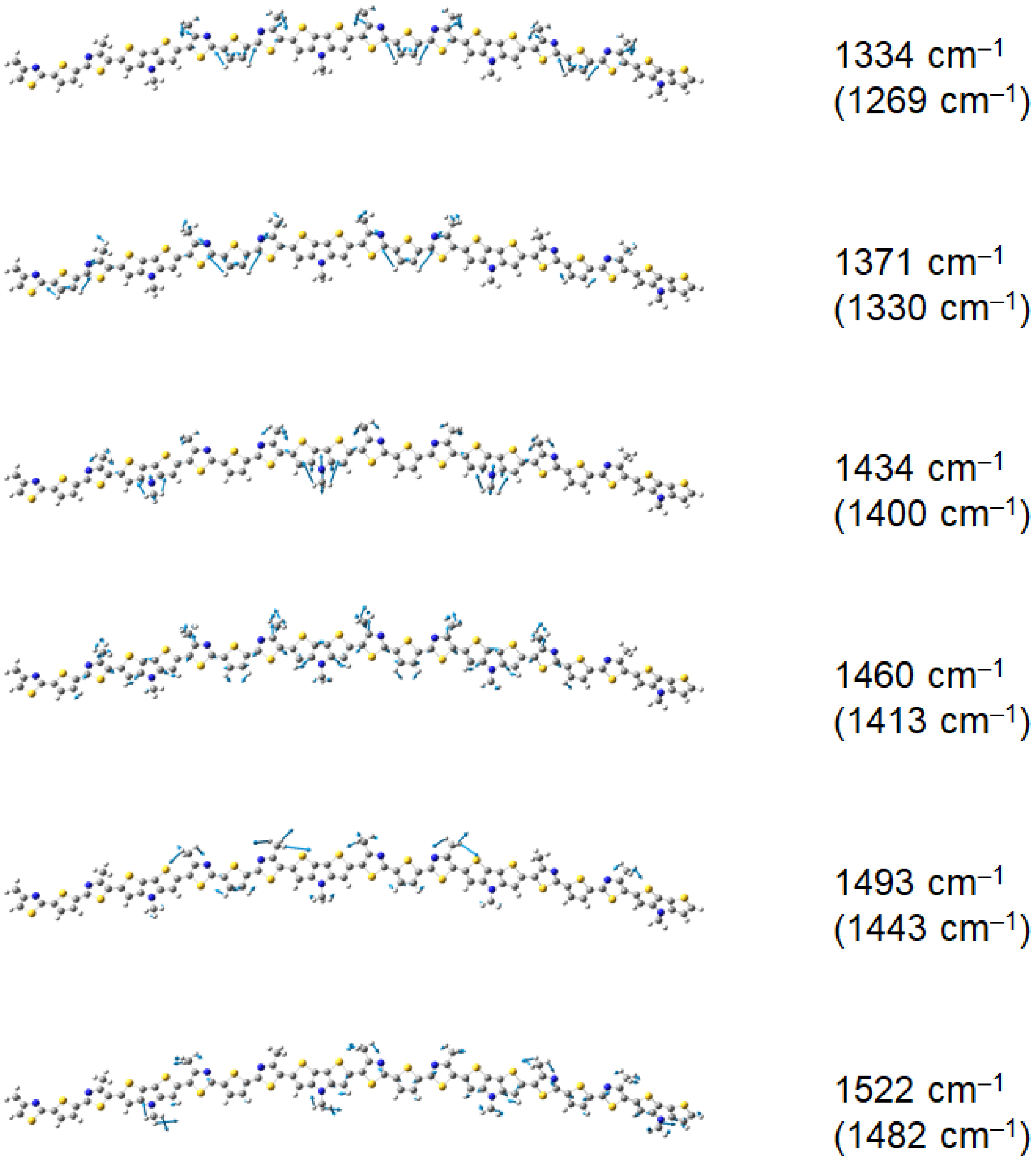

Figure S10. DFT-calculated stretching modes of PDTz tetramer. The wavenumbers in parentheses indicate experimental values.

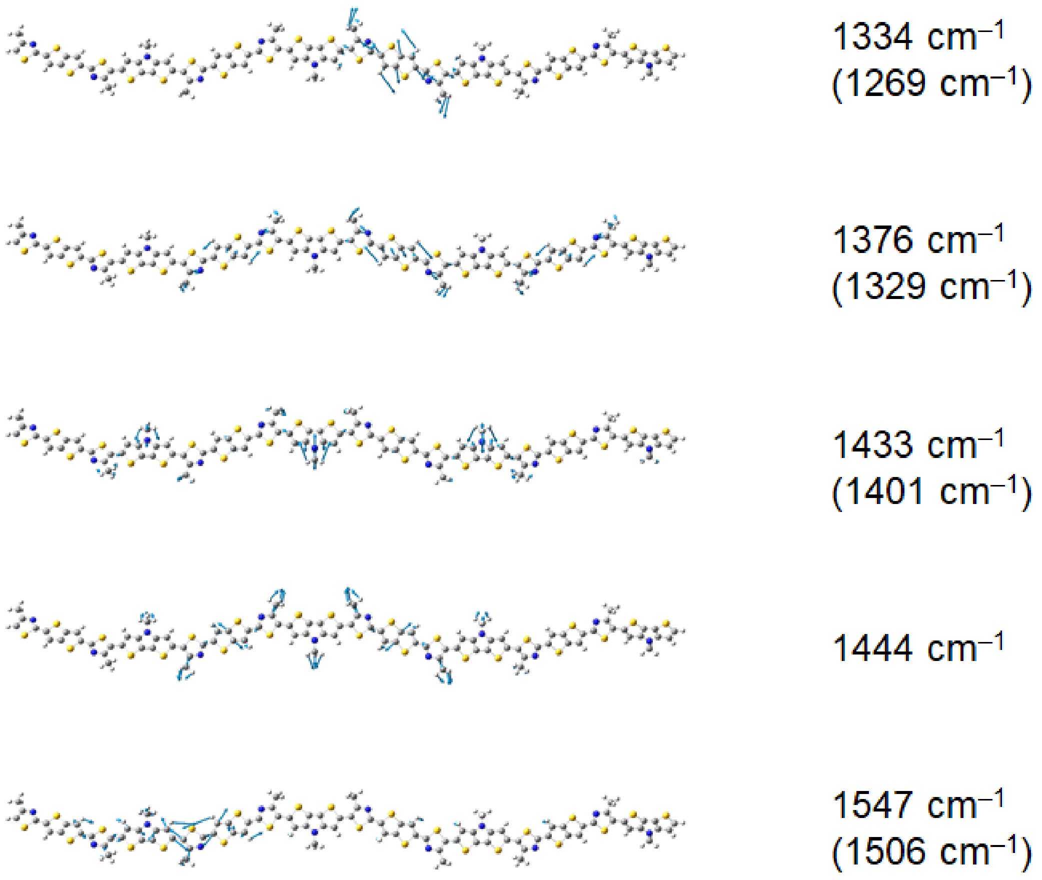

Figure S11. DFT-calculated stretching modes of PDTTz tetramer. The wavenumbers in parentheses indicate experimental values.

Table S1. d-spacing of (100) and (010) peaks of **Figure 5c-f**.

F4TCNQ conc. (mg·mL^{-1})	PDTz		PDTTz	
	(100) d-spacing (Å)	(010) d-spacing (Å)	(100) d-spacing (Å)	(010) d-spacing (Å)
0.0	15.14	3.71	14.84	3.69
0.5	16.39	3.65	15.86	3.64
1.0	16.58	3.65	16.05	3.65
1.5	16.55	3.65	16.06	3.63
2.0	16.63	3.65	16.06	3.63
